# Genetic Ancestry Reveals Historical Diversity of Formation Across Three Brazilian Communities of African Descent (*Quilombos*) in Central Brazil

**DOI:** 10.1002/ajhb.70199

**Published:** 2026-01-23

**Authors:** Sabrina Guimarães Paiva, Anna C. Rivara, Matheus de Castro Nóbrega, Rafaela de Cesare Parmezan Toledo, Maria de Nazaré Klautau‐Guimarães, Sidney Emanuel Batista dos Santos, Lorena Madrigal, Silviene Fabiana de Oliveira

**Affiliations:** ^1^ Departamento de Genética e Morfologia, Instituto de Ciências Biológicas Universidade de Brasília Brasília Distrito Federal Brazil; ^2^ Programa de Pós‐Graduação Em Biologia Animal, Instituto de Ciências Biológicas Universidade de Brasília Brasília Distrito Federal Brazil; ^3^ Instituto Federal de Educação Ciência e Tecnologia Do Tocantins Araguaína Tocantins Brazil; ^4^ Programa de Pós‐Graduação (Mestrado) em Demandas Populares e Dinâmicas Regionais (PPGDire) Universidade Federal Do Norte Do Tocantins Araguaína Tocantins Brazil; ^5^ Department of Social and Behavioral Sciences Peter O'donnell School of Public Health University of Texas Southwestern Medical Center Dallas Texas USA; ^6^ Programa de Pós‐Graduação Em Ensino de Biologia (PROFBIO), Instituto de Ciências Biológicas Universidade de Brasília Brasília Distrito Federal Brazil; ^7^ Instituto de Ciências Biológicas, Programa de Pós‐Graduação Em Genética e Biologia Molecular (PPGBM), Laboratório de Genética Humana e Médica (LGHM) Universidade Federal do Pará (UFPA) Belém Pará Brazil; ^8^ Department of Anthropology University of South Florida Florida USA

## Abstract

**Introduction:**

Characterized as relatively isolated communities, many Brazilian *quilombos* were formed during the period of slavery in Brazil when enslaved persons (most of African descent) ran away or were abandoned by their enslavers. *Quilombos* in Central Brazil, whose settlement was more recent due to the relative isolation of the region, remain understudied. To address this gap, this study estimated the genetic ancestry of three *quilombo* communities in Central Brazil.

**Methods:**

A cross‐sectional study was performed among three Central Brazilian *quilombos*, Cocalinho (*N* = 54) and Pé do Morro (*N* = 58) located in the Brazilian state of Tocantins, and Kalunga (*N* = 132) located in the state of Goiás. Genetic ancestry was estimated from 61 Ancestry‐informative INDEL biallelic markers collected from blood samples and analyzed using STRUCTURE v 2.3. Statistical analyses were performed using SAS statistical software, v. 9.4.

**Results:**

The population demonstrated heterogeneous genetic admixture by *quilombo*. Average African admixture estimates were 36.75%, 29.82%, and 63.17% in Cocalinho (semirural), Pé do Morro (urban), and Kalunga (rural) communities, respectively. Indigenous and European ancestry contributions also varied by *quilombo*, with participants from the more recently populated *quilombos* and those living closest to urban areas having higher European and Indigenous genetic ancestry contributions.

**Conclusions:**

This study demonstrates that *quilombos* comprise rich population histories shaped by culture, historical events, and sociodemographic and environmental interactions. By unraveling the genetic tapestry of Central Brazil's *quilombos*, this study contributes to a deeper understanding of Brazil's intricate social and historical landscape.

## 1 Introduction

Brazil is well known for having a highly multiethnic population, resulting from an intense process of genetic admixture between three main parental groups: Indigenous, sub‐Saharan African and European—mostly Iberians (Salzano and Sans 2014; Moura et al. 2015; Pena et al. 2020). However, the genetic contributions of these three groups throughout the Brazilian territory were not homogeneous and the proportion of genomic ancestry from these populations differs significantly by geographic region (Santos et al. 2010; Pena et al. 2011; Manta et al. 2013; Moura et al. 2015; Resque et al. 2016; Salzano and Sans 2014; Souza et al. 2019). Presently, among the general Brazilian population, European genetic ancestry tends to be higher in the South, Southeast and Central West regions (in that order); African ancestry tends to be higher in the Northeast, and Indigenous ancestry is lower in the South, Southeast and Northeast, compared to the Central West and North regions (Pena et al. 2011; Salzano and Sans 2014; Kehdy et al. 2015). There are exceptions to this pattern, as observed among Indigenous populations (Castilla and Schuler‐Faccini 2014), and *quilombo* populations; the latter is unique for their higher degree of African ancestry irrespective of their geographic locale (Pedrosa 2006; Luizon et al. 2008; Scliar et al. 2009; Santos et al. 2010).

The formation of *quilombo* communities was a consequence of the Transatlantic Slave Trade. Historically, Africans were first brought to Brazil as enslaved persons (slavery was legal in Brazil between the early to mid‐1500s to 1888). *Quilombos*, characterized as relatively isolated communities, were formed when some of those enslaved persons ran away or were abandoned by their enslavers (Paiva et al. 2020; Joerin‐Luque et al. 2023).

Despite being regarded as relatively isolated communities, *quilombos* have always maintained commercial and social interactions with surrounding populations, resulting in the evident admixture that has occurred since their formation (Amorim et al. 2011; Gontijo et al. 2018). In addition to the *quilombos* established during the slavery period, many also formed after the formal abolition of slavery (1888), as this form of community organization continued to be, for many of them, the only possibility to live in freedom and social integration (Matos and Eugenio 2018). For the first time in Brazilian history, the 2022 Census acknowledged the existence of the *quilombo* population. Census results showed that the *quilombo* population comprises approximately 1.32 million people, or 0.65% of the country's total inhabitants. Presently, some 3752 communities are certified as being *quilombo* throughout Brazil (Fundaçao Cultural Palmares 2024); however, only 494 of them have been legally recognized and had their land rights upheld by the government (IBGE 2023), and 4.3% of the *quilombo* population resides in legally recognized *quilombo* communities (IBGE—https://www.ibge.gov.br/).

Previous studies of the genetic diversity and ancestry of *quilombos* have analyzed small sets of classical autosomal markers or focused on mtDNA and Y‐chromosome variation (as reviewed in Salzano and Sans 2014; Resque et al. 2016; Souza et al. 2019; Nunes et al. 2020; Joerin‐Luque et al. 2023). These studies showed ancestry patterns consistent with *quilombos*' known history and indicated that African genetic ancestry was the predominant genomic component, with variable and smaller proportions of European and Indigenous coancestry being noted (Amorim et al. 2011; Lopes Maciel et al. 2011; Kimura et al. 2013; Gontijo et al. 2018), although some notable exceptions have been found (Lopes Maciel et al. 2011; Kimura et al. 2013; Gontijo et al. 2014).

Ancestry inference in populations with a recent history of admixture, such as those in Brazil, remains a challenge (Escher et al. 2022; De Oliveira et al. 2023). Using a set of 40 INDEL polymorphisms, Pena et al. (2011) assessed 934 individuals (self‐ascribing as White, Brown, or Black) from the four most populated Brazilian regions and found that European ancestry was higher and more homogeneous among participants than previously expected. However, several other studies have highlighted the genetically heterogeneous populations in Brazil, with greater diversity being observed among traditional communities such as *quilombos* (Souza et al. 2019; Joerin‐Luque et al. 2023).

The genetic ancestry of *quilombos* in Central Brazil remains understudied. Due to the large size and rich history of the Central Brazil region—and given that population history diversity is present even within smaller regions (i.e., Central America and the Caribbean) (Madrigal 2006; Nieves‐Colon 2022)—it is expected that the genetic makeup of *quilombo* communities within Central Brazil will differ from those in other regions, likely reflecting different evolutionary, ecological, and historical processes. To understand the differences in admixture among Brazilian *quilombos*, we estimated the contribution of the three ancestral groups to *quilombo* communities within Central Brazil.

## 2 Methods

### 2.1 Study Design and Populations

A cross‐sectional population‐based study was performed in three *quilombo* communities in Central Brazil. They included Cocalinho (a semirural community), Pé do Morro (an urban community), and Kalunga (a rural community). Cocalinho and Pé do Morro have approximately 700 and 600 inhabitants, respectively. Both are located in the northern part of Tocantins state. Kalunga has a population of approximately 10 000 inhabitants, and is located in Goiás state (Fernandes 2015). The Kalunga are organized into more than 20 communities that reside in 62 locations over an area of approximately 272 000 ha. All three *quilombo* communities are registered by Fundação Cultural Palmares, a Brazilian governmental organization that certifies *quilombos* in the country.

Fieldwork was conducted between May 2014 and May 2015. Greater details about the work including insights regarding the health of the communities can be found elsewhere (Paiva et al. 2022). Sample collection and analysis were approved by the Ethics Committee of the College of Health Sciences of University of Brasilia (CEP‐FS/UnB 214/13), and participants provided informed consent.

### 2.2 Sample and Data Collection

All participants were registered as members of their *quilombo* association. Inclusion criteria were being a registered member of the *quilombo*, residing in the *quilombo* community, and being ≥ 18 years old. All eligible participants were invited and received information about the objectives of the study, ensuring equal chance of participation. Exclusion criteria were being a nonregistered member, aged < 18 years, infirm or having cognitive impairment, and/or lacking independent communication skills. Additionally, we excluded closely related individuals (at least a genetic coefficient of inbreeding ≤ 1/16). A total of *N* = 244 unrelated participants of both sexes were included in the study, *N* = 54 from Cocalinho, *N* = 58 from Pé do Morro, and *N* = 132 from Kalunga. All participants were interviewed about their self‐identified ethnicity/race using the five categories (White, Black, Indigenous, Asian, Mixed) recognized by the Brazilian Institute of Geography and Statistics (IBGE 2011).

### 2.3 Genetic Ancestry Estimation

Whole blood samples (10 mL) were collected from 244 participants via a venous blood draw and stored in EDTA collection tubes. DNA was extracted from the samples using Puregene Kits (QIAGEN) and genomic DNA was quantified with a NanoDrop 1000TM. Genotyping to determine ancestry estimation was carried out using 61 INDEL‐type markers (insertion/deletion) that have been standardized and validated (Ramos et al. 2016; Andrade et al. 2018). Two multiplex PCRs were performed, followed by electrophoresis on the ABI Prism 3130 sequencer (Applied Biosystems, Life Technologies, Carlsbad, CA, USA) and analysis using the GeneMapper ID v.3.2 software (Applied Biosystems, Life Technologies, Carlsbad, CA, USA). Individual proportions of European, African, and Indigenous genetic ancestry were estimated using Structure software v.2.3.3. Estimation of the ancestry of the Brazilian samples was performed with population samples (*n* = 593) from three parental populations, as used similarly by Santos et al. (2010). These populations included Africans (from Angola, Mozambique, Zaire, Cameroon, and the Ivory Coast), Europeans (mainly Portuguese), and Native Americans (individuals from the indigenous tribes of the Brazilian Amazon region). Please note in this regard that, in studies of Brazilian genetic ancestry, Native American, Indigenous, and Amerindian are all frequently used and interchangeable terms (Santos et al. 2010; Pena et al. 2020; Nunes et al. 2025). We have chosen to use the term “Indigenous” as it is the term used by the Brazilian Institute of Geography and Statistics (and was one of the choices offered to participants when self‐identifying).

### 2.4 Statistical Analysis

The code and results for this paper were generated using SAS statistical software, v 9.4 (Copyright [2013] SAS Institute Inc., Cary, NC, USA). As the admixture model assumes that everyone inherits part of their ancestral markers from ancestral populations, the results were plotted against the three parental populations that constitute the Brazilian population to perform ancestry stratification. Structure v 2.3.4 software was used to estimate admixture with 50 000 burn length, 100 000 MMC repetitions after burning, in allele frequencies independent model. Data were normalized and then analyzed by MANOVA using the software SPSS 22 (IBM SPSS 22.0 Statistics for Windows Armonk (NY): IBM Corp; 2013). We estimated genetic differentiation between populations (FST) and genomic ancestry using Arlequin v. 3.5.

## 3 Results

The sociodemographic characteristics of the participants are summarized in Table 1. The mean age was 52.7 (± 14.6) years for Cocalinho (*n* = 54), 61.5 (± 10.9) years for Pé do Morro (*n* = 58), and 56.7 (± 11.7) years for Kalunga (*n* = 132). Most participants were women. In Cocalinho and Kalunga, most individuals self‐ascribed as Black and comprised 57.4% and 54.5% of the respective populations. Participants from the Pé do Morro community predominantly self‐ascribed as Mixed‐race.

**TABLE 1:** Sociodemographic data of participants included in the study.

Variables	Cocalinho (*N* = 54)	Pé do Morro (*N* = 58)	Kalunga (*N* = 132)
Age (years)	52.70 ± 14.65	61.58 ± 10.98	56.76 ± 11.72
Sex	*N* (%)	*N* (%)	*N* (%)
Male	23 (42.6)	26 (44.8)	65 (49.2)
Female	31 (57.4)	32 (55.2)	67 (50.8)
Self‐identified race/ethnicity			
White	6 (11.1)	6 (10.3)	2 (1.5)
Black	31 (57.4)	19 (32.8)	72 (54.5)
Indigenous	2 (3.7)	—	1 (0.8)
Mixed	14 (25.9)	33 (56.9)	55 (42.3)
Not declared	1 (1.9)	—	2 (1.5)

*Note:* Variable age presented as mean (±SD). Others variables presented as percentage (total number).

**FIGURE 1:**
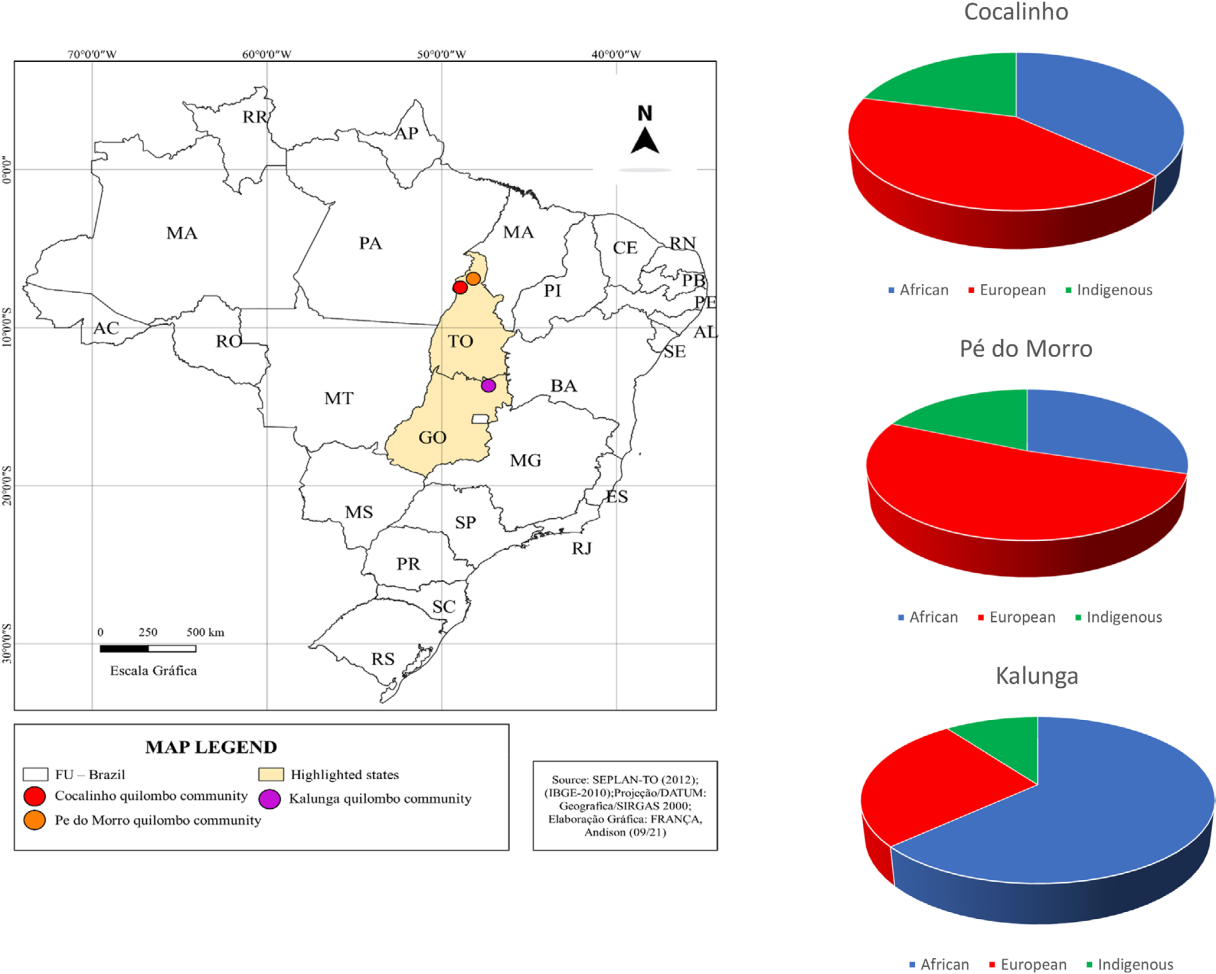
Map of Brazil indicating the location of the three Quilombos (Cocalinho, Pé do Morro and Kalunga) and pie charts showing their population ancestry estimates. Analysis was done on STRUCTURE 2.3.

Mean global African, European, and Indigenous genetic contributions are shown in Figure 1. The Kalunga community had greater African genetic ancestry contributions compared to contributions from Indigenous and European origins. Cocalinho and Pé do Morro had a higher proportion of European genetic ancestry in relation to the other ancestries. The smallest genetic contribution found among the three communities was Indigenous (the largest Indigenous contribution being found in Cocalinho).

**FIGURE 2:**
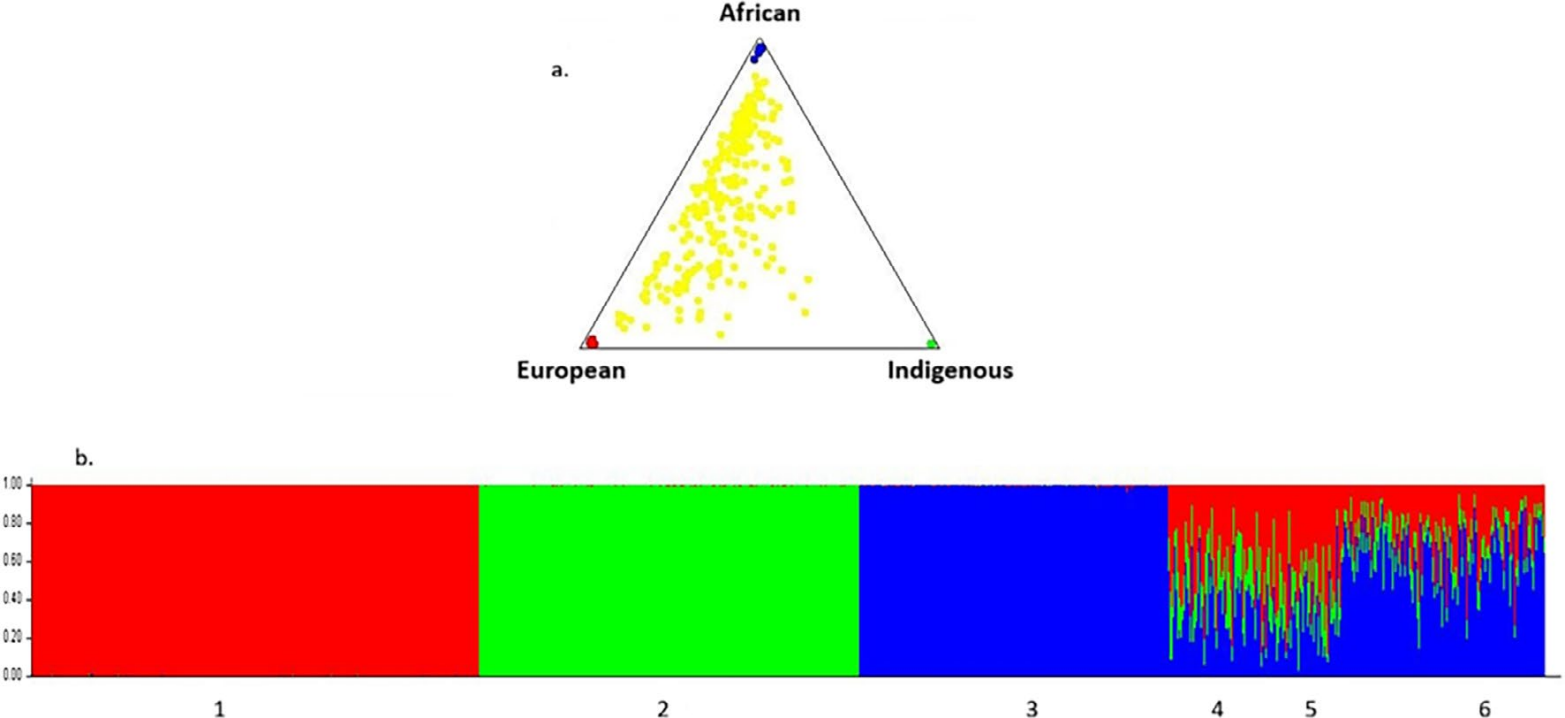
(a) Triangular diagram of the genetic structuring analysis obtained using the STRUCTURE v. 3.2 program. Each yellow dot represents an individual that was classified and plotted according to the estimate of individual admixture based on the INDEL type genetic markers evaluated. Colors Red = European parent; Blue = African parent; Green = Indigenous parent; Yellow = samples from the three *quilombo* populations analyzed as a single population. The figure shows a greater concentration of yellow dots (individuals) close to African and European ancestries. (b) Graphical representation of the genetic structure of the parental and quilombo populations obtained from the STRUCTURE v. 3.2 program. (1) European parental, (2) Indigenous parental, (3) African parental, (4) 54 individuals from the Cocalinho *quilombo* community, (5) 58 individuals from the Pé do Morro quilombo community, and (6) 132 individuals from the Kalunga *quilombo* community.

The ternary graphic representation (Figure 2a) shows the distribution of the estimated percentage of the genetic contribution of each ancestry (African, Indigenous and European); each vertex of the triangle represents a parental subpopulation. The individuals from the three newly analyzed *quilombo* communities (yellow dots) were plotted in the triangle according to the Bayesian classification, being considered as a single population. Each dot represents an individual who was classified and plotted according to the estimate of individual admixture, based on the INDEL type genetic markers evaluated. This representation indicates that the individuals from the three *quilombo* communities presented a tri‐hybrid genetic composition, with considerable heterogeneity and higher African and European contributions and lower Indigenous components in all three of them.

Figure 2b reaffirms and expands on these findings. The ancestry estimates of individuals from the three parental populations (African, Indigenous, and European) and individuals from the *quilombo* communities in this study are shown. The horizontal bar is composed of thin vertical lines, each line representing an individual. Participants' ancestry from the three parental populations [European (1), Indigenous (2), and African (3)] was classified and plotted on the graph according to genetic structure. The numbers 4, 5, and 6 represent the individuals from the *quilombo* communities analyzed (Cocalinho, Pé do Morro, and Kalunga, respectively). Again, the results demonstrate tri‐hybrid ancestry and reveal genetic ancestry contributions from all three parental populations, but in different proportions by *quilombo*. These estimates show that *quilombo* communities are genetically heterogeneous. Greater African genetic contributions are observed in the Kalunga (6), and greater European genetic contributions are found in the Cocalinho (4) and Pé do Morro (5) *quilombo* communities.

The measures of genetic differentiation (Fst) calculated between the Cocalinho, Pé do Morro and Kalunga communities were statistically significant (*p* ≤ 0.05) in all analyses, indicating differences between the gene pools of the three populations. Cocalinho and Pé do Morro had the lowest degree of genetic differentiation, while the Fst value between Kalunga and Pé do Morro was higher (> 5%), indicating greater differentiation.

The distribution of individual ancestries in the study populations is shown in Table 2. Half of the sample from Cocalinho carried more than 40% of European contribution while the remaining half had more than 40% African ancestry. In Pé do Morro, 70.68% of the individuals had a higher European contribution. In Kalunga, more than 90% of the individuals were observed with African contributions above 40%, with no high Indigenous contributions observed.

**TABLE 2:** Distribution of individual ancestries in the study populations.

Ancestry	Cocalinho (*N* = 54)	Pé do Morro (*N* = 58)	Kalunga (*N* = 132)
African			
Minimum	5.90	3.20	14.50
Maximum	72.40	79.10	90.00
Median	37.40	27.20	66.15
Mean ± SD	36.75 ± 17.12	29.82 ± 15.82	63.17 ± 15.95
European			
Minimum	10.70	12.70	4.60
Maximum	84.20	87.90	73.20
Median	41.05	51.40	24.10
Mean ± SD	42.38 ± 18.19	51.69 ± 18.58	26.59 ± 14.43
Indigenous			
Minimum	6.20	3.70	3.30
Maximum	53.00	57.70	36.40
Median	21.25	17.30	8.60
Mean ± SD	20.87 ± 10.65	18.49 ± 10.78	10.24 ± 5.45

*Note:* Variable ancestry presented as mean (±SD). This data was taken from the general spreadsheet generated by the Structure program.

Genetic ancestry and different categories of self‐ascribed race/ethnicity in the study populations are shown in Table S1. A forthcoming paper will examine associations between self‐ascribed ethnicity and the genetic ancestry estimates obtained in these samples. For this reason, we do not discuss these results any further.

## 4 Discussion

Here, we present one of the first studies to assess the genetic ancestry of *Quilombo* communities within central Brazil, and specifically the states of Tocantins and Goiás. These areas have been historically understudied in terms of genetic history and admixture (Gontijo et al. 2018), in comparison to urban centers (Giolo et al. 2012; Pereira et al. 2024) and other rural *quilombo* communities in Brazil (Kimura et al. 2013; Gontijo et al. 2018; Nunes et al. 2020). The results demonstrate that the three populations were genetically admixed, with each showing different patterns of ancestry. Participants from the most recently populated regions and those living closest to urban areas (Cocalinho and Pé do Morro) showed higher European genetic contributions, whereas African ancestry contributions were higher in the more rural locale (Kalunga). Ethnic/ancestry self‐assignment also differed among individuals from the communities.

The results indicate that the composition of the communities in Tocantins is genetically similar to other *quilombo* communities located in North and Southeast regions (Amorim et al. 2011; Lopes Maciel et al. 2011; Kimura et al. 2013). The ancestry of the Kalunga, who showed significant genetic differentiation from the Cocalinho and Pé do Morro communities, has greater genetic similarity to individuals from other rural *quilombos* (Lopes Maciel et al. 2011; Amorim et al. 2011; Kimura et al. 2013).

The Kalunga, a community occupying a large territory, had the largest African contribution in the present study, as found previously (Gontijo et al. 2018). Interestingly, the African genetic contribution was higher compared to those of other *quilombo* communities previously studied in Brazil (Vallinoto et al. 2003; Santos et al. 2010; Lopes Maciel et al. 2011; Amorim et al. 2011; Kimura et al. 2013; Gontijo et al. 2014). This finding could be due to the Kalunga's geographical distance from urban centers (Meegen‐Silva 1999; Paiva et al. 2022) and/or the tendency for Brazilians to marry individuals of similar ancestry as revealed by data from the EPIGEN‐Brazil project (Tarazona‐Santos et al. 2016). This scenario of ethnic‐racial mediated marriages may be due to the various phenotypic, cultural, and social characteristics interconnected with ancestry (Kehdy et al. 2015). Similar behavior, compounded with the relative isolation of this community, may explain some of the reduced heterogeneity in ancestry observed among the Kalunga.

The other two communities (both within Tocantins) had higher European genetic contributions, possibly reflecting the area's history, geographic proximity, and easier access to urban centers (Paiva et al. 2022). Additionally, the formation of Tocantins' *quilombo* communities intersects with the migratory movements of several families from the Brazilian Northeast region, mainly from the state of Maranhão, at different times during the 1960s to the 1990s (Oliveira 2021). This movement was largely triggered by climatic and economic conditions (Paiva et al. 2020), and included the families who arrived in Morro Santo, the region where the Pé do Morro community is currently located (Oliveira 2021). Historically, Maranhão was characterized by having many *quilombos* and slave‐owning families. These ancestries are reflected in their descendants who migrated to Tocantins (Paiva et al. 2020). This may partially explain the lower estimates of genetic differentiation observed between Pé do Morro and Cocalinho.

The lowest ancestry contribution found among all three communities was Indigenous, even though two of the communities are located in close proximity to the North (the region with the highest Indigenous genetic contribution in relation to other Brazilian regions) (Salzano and Sans 2014). In a study conducted by Gontijo et al. (2014) using 15 autosomal ancestry informative markers (AIMs), other *quilombo* communities in northern regions of Brazil had higher estimates of Indigenous ancestry (more than 40%). However, the results here are similar to those found in other studies within Brazil that used mitochondrial and Y chromosome markers and showed high female contributions of African origin (Bortolini et al. 1999; Ferreira 2006; Carvalho et al. 2008), high male contributions of European and African ancestry, and lower contributions of Indigenous ancestry (Bortolini et al. 1999; Ribeiro‐dos‐Santos et al. 2002; Ribeiro et al. 2009, 2011; Kimura et al. 2017). Other assessments using AIMs have also estimated high European and African parental contributions and variable but generally lower Indigenous contributions (Bortolini et al. 1999; Vallinoto et al. 2003; Pedrosa and Oliveira 2004; Pedrosa 2006; Luizon et al. 2008; Amorim et al. 2011), reflecting the complexity and heterogeneity in the history of formation and admixture of the Brazilian population (Tarazona‐Santos et al. 2016).

Indigenous contributions have also been found to be generally low among *quilombos* communities living in urban areas (Chor et al. 2019; Pena et al. 2020). These studies revealed that higher European genetic ancestry characterizes these communities (Manta et al. 2013; Pereira et al. 2024; Callegari‐Jacques et al. 2003; Parra et al. 2003; Bomfim 2008; Moura et al. 2015). Our findings support these patterns.

As noted above, *Quilombo* communities are generally viewed as being relatively isolated communities, characterized by histories of resistance to oppression and the formation of communities of refuge in hard‐to‐reach places (Lemes et al. 2018). However, the influx of historical migration into these areas has partially dissolved these barriers, which is evidenced by the patterns of admixture observed within these communities (Lemes et al. 2018; De Oliveira et al. 2023; ***this study). As our results show, the formation and population history of African‐derived communities, such as *quilombos*, in the Americas was a complex and context‐specific process, likely affected by migration, social, economic, and political conditions, and by the local ecology (Madrigal 2006).

### 4.1 Self‐Identification

Our results indicate discordance between self‐assignment and genetic ancestry and show that self‐assignment of ethnicity/ancestry is complex and demands further investigation (Madrigal et al. in progress). Most Brazilians have a high degree of European ancestry (Pena et al. 2020), a significant degree of African ancestry, and a significant and similar degree of Indigenous ancestry (Pena et al. 2020). Our study demonstrates that these ancestries are also represented in self‐assignment but are heterogeneous in the degree of representation among *quilombo* communities in Central Brazil.

Societal conditions have and continue to shape how people self‐assign their race or ethnicity. Recent affirmative actions and public policies implemented in Brazil, with the intention of promoting equal opportunities for individuals of all ethnic‐racial categories over the last decade, have contributed to greater acknowledgement and recognition of Brazil's rich genetic diversity and history. This may have influenced how or why participants' ethnicity/ancestry self‐assignments differed.

### 4.2 Implications for Dissemination and Future Work

We are concerned about the potential oversimplification and misinterpretation of the findings. In the past, misinterpretations of genetic ancestry have been used to argue against affirmative action and public policies (Kent et al. 2014). In fact, a study by Pena et al. (2011) (regarding the high degree of European ancestry found in Brazil) has been reported several times in a sensationalist manner and has been used by others to contribute to the deconstruction of the Afro‐Brazilian identity (Kent et al. 2014). It is worth noting that erroneous, simplistic interpretations, and/or misinterpretation of research of this kind can be used to suggest that only the genetic determinism of identity should be decisive in the formulation of policies and affirmative actions. However, the degree of one's genetic ancestry may not align with their present or their ancestors' historical experiences or marginalization.

Our data, as well as the discussion addressed by Kent et al. (2014), highlights the debate over genetic determinism in Brazilian society, in our case expanding to traditional communities. Our study, as well as studies carried out by various population genetics groups, including Pena et al. (2011), enriches the information present in historical records. These studies show that perceptions of ethnic‐racial identity and genetic ancestry are dynamic and vary according to geographic location and other factors, such as social, economic, political conditions, cultural history, and individual experiences. This study highlights the importance of using a multifaceted and more nuanced approach that helps promote *quilombo* recognition beyond only genomic ancestry. An approach that also incorporates cultural traditions, oral records, religious beliefs, rituals, kinship, and shared histories and connections with territories and land cultivation (Marques and Gomes 2013; Farfán‐Santos 2015; Brazil 2006) may better capture the storied experiences of *quilombo* communities.

### 4.3 Strengths and Limitations

Our study provides much needed insight into the population history of *quilombos* in Central Brazil. We were able to show similarities between the genetic composition of the Kalunga *quilombo* to other rural *quilombos* and similarities between the genetic composition of Pé do Morro and Cocalinho to other more urban *quilombos* throughout the country. Notably, we provide further support that *quilombos* are diverse communities, with differing admixture estimates from African, European, and Indigenous ancestries.

This study is limited in several ways. Differing methods were used to assess ancestry among the quilombos, which limits our ability to compare among them, as well as compare them to others. A range of genetic marker panels has also been used in the study of Brazilian populations (Escher et al. 2022). It is noteworthy that the Kalunga community itself has been studied using different genetic markers including AIM and STR (Pedrosa 2006), mtDNA (Ferreira 2006), Y chromosome (Ribeiro 2009), and Alu insertions (Silva 2016). The precision of these estimates is directly proportional to the number of markers used and the magnitude of the difference in their allele frequencies (Shriver and Kittles 2004; Santos et al. 2016), especially when considering Indigenous ancestry. Therefore, the accuracy of these estimates is directly related to the number of markers analyzed; the greater the number, the more regions of the genome are analyzed (Shriver and Kittles 2004). We are limited in our capacity to estimate genetic ancestry and make comparisons to other groups due to the methodologies used, and the small sample size and representativeness of our studied participants (Salzano and Sans 2014; Madrigal and Barbujani 2007). As in other studies, the use of different markers along with the use of different population sample sets limits comparisons between the participants here and those assessed in other studies (Joerin‐Luque et al. 2023).

## 5 Conclusions

Our study provided insight into the genetic ancestry of *quilombo*s in Central Brazil. The results support that *quilombo* communities have diverse and rich population histories and share cultural histories of social and economic vulnerability and resistance. By unraveling the genetic tapestry of Central Brazil's *quilombos*, our study contributes to a deeper understanding of Brazil's intricate social and historical landscape.

## Author Contributions

**Sabrina Guimarães Paiva:** conceptualization (lead), formal analysis, investigation (fieldwork), methodology, resources, writing – original draft preparation, and writing – review and editing. **Anna C. Rivara:** writing – review and editing. **Matheus de Castro Nóbrega:** investigation (fieldwork) and resources (supporting). **Rafaela de Cesare Parmezan Toledo:** investigation (fieldwork), resources (supporting), and writing – review and editing. **Maria de Nazaré Klautau‐Guimarães:** conceptualization, funding acquisition (lead), management and coordination responsibility for the research activity planning and execution, resources, and supervision (supporting). **Sidney Emanuel Batista dos Santos:** lab analysis (lead), resources (supporting), and writing – review and editing. **Lorena Madrigal:** formal analysis (lead), methodology, writing – review and editing, and supervision (supporting). **Silviene Fabiana de Oliveira:** conceptualization (lead), funding acquisition, investigation (fieldwork), management and coordination responsibility for the research activity planning and execution, project administration, resources, and supervision (lead). All authors reviewed and approved the final submission.

## Funding

Conselho Nacional de Desenvolvimento Científico e Tecnológico (CNPq), Coordenação para Aperfeiçoamento de Pessoal de Nível Superior (CAPES) e Decanato de Extensão, Universidade de Brasília, Brazil.

## Conflicts of Interest

The authors declare no conflicts of interest.

## Supporting information


**Table S1:** Genetic ancestry in different categories of self‐identified race/ethnicity in the study populations.

## Data Availability

The data that support the findings of this study are available on request from the corresponding author. The data are not publicly available due to privacy or ethical restrictions.
