# Supplementary material for: Genetic Ancestry Reveals Historical Diversity of Formation Across Three Brazilian Communities of African Descent (Quilombos) in Central Brazil
Source: Am J Hum Biol. 2026 Jan 23;38(1):e70199. doi: 10.1002/ajhb.70199 (PMC12828477; doi:10.1002/ajhb.70199)
Supplement: Supplementary file 1 — Table S1: Genetic ancestry in different categories of self‐identified race/ethnicity in the study populations. [file AJHB-38-e70199-s001.docx]

**Supplementary Table 1: Genetic ancestry in different categories of self-identified race/ethnicity in the study populations**

|  | **Self-identified race/ethnicity** | | | | | | | | | | | | |
| --- | --- | --- | --- | --- | --- | --- | --- | --- | --- | --- | --- | --- | --- |
| **Ancestry** | **White** | | | **Black** | | | **Indigenous** | | | **Mixed** | | |  |
| **Cocalinho** | Minimum | Median | Maximum | Minimum | Median | Maximum | Minimum | Median | Maximum | Minimum | Median | Maximum |  |
| African | 6.0 | 29.1 | 48.0 | 8.0 | 44.6 | 72.0 | 43.0 | 44.6 | 46.0 | 9.0 | 25.2 | 45.0 |  |
| European | 38.0 | 46.9 | 81.0 | 11.0 | 32.5 | 84.0 | 30.0 | 32.4 | 35.0 | 22.0 | 53.8 | 76.0 |  |
| Indigenous | 6.0 | 13.9 | 35.0 | 7.0 | 20.8 | 53.0 | 23.0 | 23.1 | 24.0 | 7.0 | 22.5 | 36.0 |  |
| **Pé do Morro** |  |  |  |  |  |  |  |  |  |  |  |  |  |
| African | 3.0 | 16.6 | 43.0 | 15.0 | 27.3 | 56.0 | - | - | - | 6.0 | 29.6 | 79.0 |  |
| European | 40.0 | 68.3 | 88.0 | 31.0 | 48.9 | 75.0 | - | - | - | 13.0 | 51.8 | 87.0 |  |
| Indigenous | 5.0 | 15.1 | 37.0 | 5.0 | 17.8 | 39.0 | - | - | - | 4.0 | 17.2 | 58.0 |  |
| **Kalunga** |  |  |  |  |  |  |  |  |  |  |  |  |  |
| African | 26.0 | 36.9 | 48.0 | 37.0 | 67.1 | 90.0 | - | - | - | 15.0 | 60.5 | 88.0 |  |
| European | 39.0 | 46.8 | 55.0 | 5.0 | 20.8 | 46.0 | - | - | - | 5.0 | 30.3 | 73.0 |  |
| Indigenous | 14.0 | 16.4 | 19.0 | 3.0 | 8.7 | 36.0 | - | - | - | 4.0 | 8.4 | 26.0 |  |
